# The biological role of the medial olivocochlear efferents in hearing: separating evolved function from exaptation

**DOI:** 10.3389/fnsys.2015.00012

**Published:** 2015-02-25

**Authors:** David W. Smith, Andreas Keil

**Affiliations:** ^1^Program in Behavioral and Cognitive Neuroscience, Department of Psychology, University of FloridaGainesville, FL, USA; ^2^Center for Smell and Taste, University of FloridaGainesville, FL, USA; ^3^Center for the Study of Emotion and Attention, University of FloridaGainesville, FL, USA

**Keywords:** medial olivocochlear efferents, MOC, signal-to-noise ratio, protection from acoustic trauma, outer hair cells, OHC, corticofugal pathways, auditory attention

## Abstract

Cochlear outer hair cells (OHCs) are remarkable, mechanically-active receptors that determine the exquisite sensitivity and frequency selectivity characteristic of the mammalian auditory system. While there are three to four times as many OHCs compared with inner hair cells, OHCs lack a significant afferent innervation and, instead, receive a rich efferent innervation from medial olivocochlear (MOC) efferent neurons. Activation of the MOC has been shown to exert a considerable suppressive effect over OHC activity. The precise function of these efferent tracts in auditory behavior, however, is the matter of considerable debate. The most frequent functions assigned to the MOC tracts are to protect the cochlea from traumatic damage associated with intense sound and to aid the detection of signals in noise. While considerable evidence shows that interruption of MOC activity exacerbates damage due to high-level sound exposure, the well characterized MOC physiology and evolutionary studies do not support such a role. Instead, a MOC protective effect is well explained as being a byproduct of the suppressive nature of MOC action on OHC mechanical behavior. A role in the enhancement of signals in noise backgrounds, on the other hand, is well supported by (1) an extensive physiological literature (2) examination of naturally occurring environmental acoustic conditions (3) recent data from multiple laboratories showing that the MOC plays a significant role in auditory selective attention by suppressing the response to unattended or ignored stimuli. This presentation will argue that, based on the extant literature combining the suppression of background noise through MOC-mediated rapid adaptation (RA) with the suppression of non-attended signals, in concert with the corticofugal pathways descending from the auditory cortex, the MOC system has one evolved function—to increase the signal-to-noise ratio, aiding in the detection of target signals. By contrast, the MOC system role in reducing noise damage and the effects of aging in the cochlea may well represent an exaptation, or evolutionary “spandrel”.

## Introduction and overview

The exquisite sensitivity and frequency selectivity characteristic of the mammalian auditory system are consequences of the active mechanical behavior of cochlear outer hair cells (OHCs). Driven by the largest membrane potential in the body, OHCs increase the amplitude of the basilar membrane traveling wave by two to three orders of magnitude in response to sound. The main responses of OHCs are not thought to be communicated directly to the auditory CNS via afferent relays, but instead are reflected in the response of inner hair cells, and their rich primary afferent innervation, through the resulting mechanical disturbances in the Organ of Corti and subtectorial fluids.

OHCs, for their part, are under the control of a complex descending innervation, originating from medial olivary complex in the brainstem. These medial olivocochlear (MOC) neurons can be subdivided into ipsilaterally-activated “crossed” MOC, and the contralaterally-activated “uncrossed” MOC neurons (Warr, 1992). Additionally, corticofugal pathways descend from auditory cortex to the olivary complex, and influence OHC sensitivity through MOC connections (Khalfa et al., 2001; Perrot et al., 2006; Delano et al., 2007; Schofield, 2010). Activation of the MOC in any form, by ipsilateral or contralateral sound, by electrical stimulation of either subgroup or the auditory cortex or through manipulations of attention, reduces OHC motility through a shunting of the receptor current (Rabbitt et al., 2009); reductions in auditory activity are then observed at all levels within the afferent pathways.

There remains considerable discussion concerning the role of the MOC system in hearing (cf., Kirk and Smith, 2003; Robles and Delano, 2008; Robertson, 2009; Guinan, 2010). Different functions have been proposed, with protection from noise trauma (cf., Rajan, 1988; Kujawa and Liberman, 1997; Maison et al., 2013) and the enhancement of target signals in noise (c.f., Winslow and Sachs, 1987, 1988; Kawase and Liberman, 1993; Kirk and Smith, 2003; Andéol et al., 2011) being most frequently mentioned. Here we will argue, as we have done previously (Kirk and Smith, 2003), that a meaningful discussion of *the* role of the MOC must first separate what the MOC *can be made to do*, from what it *does do*—which functions are supported by the MOC’s physiologic characteristics and by consideration of the evolutionary forces involved in shaping them. The important reasons for making this distinction lay in the resulting experimental questions and stimulus paradigms used to study the system. For example, literature searches on google scholar with search terms of “medial olivocochlear” AND “acoustic trauma” (in title and abstract) yielded approximately 6 times more published research reports than searching for “medial olivocochlear” AND “signal-to-noise”, “signals in noise” and related terms combined for recent years of 2005–2015. This suggests that authors have recently focused more on MOC protection against acoustic trauma, which—as we argue here—is likely an epiphenomenon (Kirk and Smith, 2003; Robertson, 2009; Maison et al., 2013; Liberman et al., 2014), compared with the role of the MOC in the detection of signals in noise—arguably the biological role of the system.

We approach this review from an evolutionary biological perspective because the role of the MOC must be rooted in physical conditions within naturally occurring acoustic environments and how those interact with an animal’s phenotype to determine survival. In the case of the MOC, the conditions must necessarily be universal because of the near ubiquity of the MOC across the class Mammalia. There are many studies showing that interruption of MOC function exacerbates noise-induced trauma, and we suggest that this result is a real, predictable outcome of the suppressive nature of the MOC on cochlear mechanics. Yet, the demonstration of an MOC “protective” effect requires sound pressure levels (SPL) that are extremely rare, or do not occur at all, in natural acoustic environments. This effect may thus be regarded an exaptation or evolutionary “spandrel” in the sense proposed by Gould (1997).^1^

In this review, we use our earlier paper, Kirk and Smith (2003), as a starting point, and include important new data to present a single, unitary model of the biological role of the MOC system. Many of the older, specific studies mentioned here are described in more detail in Kirk and Smith (2003). Complete references for those studies can also be found in that work but, for the sake of brevity, we have not included an extensive list here. In that paper, we reviewed the extant data describing naturally-occurring acoustic environments, combined with a cladistic analysis that suggests that MOC-innervated OHCs are a general mammalian characteristic, to argue that the environmental pressures are insufficient to account for the widespread presence of the MOC. In making our arguments concerning the evolved role of the MOC in hearing, we will account for all of the different MOC subgroups, ipsilaterally- and contralaterally-activated efferents, as well as the corticofugal pathway (i.e., why the MOC is functionally connected with the auditory cortex), in a single, critical function. The conclusions we draw here, that the MOC has a single function, namely to increase the signal to noise ratio for target signals, is supported by a large literature.

## Distribution of MOC within mammals

With the exception of three species (the microchiropteran bats Hipposideros and Rhinolophus, and the blind mole rat, Spalax Ehrenbergi), the auditory end organ of all mammals is innervated by descending, efferent neurons (see Kirk and Smith, 2003 for review). MOC systems occur in four orders of placental mammals (Primates, Chiroptera, Carnivora, Rodentia) and two orders of marsupials (Dasyuromorphia and Didelphimorphia). This suggests that the MOC system is a general feature of the mammalian auditory system, which is likely to have emerged no later than at the time of divergence between marsupial and placental mammals, ca. 173 million years ago. Among mammals for which the anatomy has been described, 21 of 24 species possess cochlear OHCs that are innervated by MOC neurons. This wide distribution, across six orders and 16 families of mammals, suggests that evolutionary selection processes have favored the mammalian MOC system, despite pronounced inter-species differences in habitat and ecology.

## Naturally-occurring acoustic environments

Our review of the literature showed that the great majority of natural acoustic environments are characterized by relatively modest (<70 dB SPL) ambient noise levels (see Kirk and Smith, 2003). The literature described both biotic and abiotic noise under a wide range of environmental conditions (e.g., ground cover, vegetation, species assemblages, etc.). Primary sources of abiotic noise include wind, rain, and wave action; wind-generated noise has a characteristic spectrum, with the dominant power peaks present at frequencies below 200–500 Hz, and water-generated noise sources have similar low-frequency spectra with peaks that extend to higher frequencies, though at frequencies above 2.0 kHz, the energy present in any one particular frequency band does not exceed 20 dB SPL.

The most intense, naturally-occurring noise environments resulted from biotic causes, for example from the chorusing of insects, birds or frogs. During these events, the highest measured SPL reached approximately 92 dB SPL in an octave band centered at 2.0 kHz in a montane rainforest in Puerto Rico (Narins, 1982). It is important to point out that higher sound levels do occur under extreme conditions, such as with thunder or at the base of a waterfall, but are rare and discontinuously distributed in time and space. Some bat species also use vocalizations in echolocation that exceed 120–140 dB SPL, but these, too, are infrequent and outside the frequency range of all but a few mammals. Interestingly, of the known mammals lacking an MOC innervation of the ear, two species are microchiropteran bats (*Hipposiderous* and Rhinolophus).

## MOC protection from noise trauma

Our earlier survey of the existing MOC-noise trauma literature concluded that MOC-based protection was evident at SPLs of 100–105 dB SPL and higher; these acoustic conditions do not have an analog in nature. This literature continues to grow and, amongst the relevant, new studies, two have employed different approaches. First, Maison et al. (2013) exposed mice to a moderate-level, 84 dB SPL, noise for 1 week to produce acute, but not chronic threshold shifts in mice. Though the acoustic conditions used remain substantially outside those typically found in nature, the objective of their work was to study the role of both lateral and medial efferent tracts in reducing the effects noise-induced loss of Type I afferents. Prior to exposure, the mice underwent surgery, damaging or removing the lateral efferent tracts synapsing on primary afferents under the IHCs, or the MOC. The results of the study showed a relatively greater loss of primary afferents with the MOC lesions, likely due to the loss of OHC gain control during noise exposure.

Likewise, taking an entirely different approach, Liberman et al. (2014) showed for the first time that chronic lesion of the MOC can have damaging effects on the normal aging of the cochlea. In that study, the MOC system was surgically lesioned in CBA/CaJ mice, then left for 39 weeks to experience normal, low-level (~40–70 dB SPL) ambient noise within the animal facility. At 45 weeks of age, the resulting effect of lack of MOC feedback was an accelerated, age-related decline in brainstem responses and a decrease in afferent synapses below inner hair cells, suggesting the MOC functions to aid in normal cochlear aging.

There are, however, complications in interpreting these interesting, new data as evidence of the protective effect of the MOC. Analysis of mouse mitochondrial DNA shows that most in-bred mouse strains have descended from the same wild *Mus musculus domesticus* female (Zheng et al., 2014). In the wild, the ancestral strain, the house mouse, reaches sexual maturity at 4–6 weeks, has a reproductive life of 5–7 months, and longevity estimates range from 3 months to between 7 and 9 months, rarely lives beyond 1 year (Latham and Mason, 2004). This is in stark contrast to the longevity of in-bred strains, which can live for >2–3 years in the laboratory.^2^ The question remains, then, whether the presence of the MOC can alter the normal aging pattern given the reproductive biology of wild mice. It is important to note that, while the literature does not support an evolved MOC protective mechanism, this does in no way minimizes the importance of deliberate manipulation of the MOC as an interventional strategy to reduce acoustic trauma. Clearly, more research is needed to better understand this phenomenon and how it might be exploited.

## Detection of signals in noise

As we pointed out above, given the wide distribution of the MOC within Mammalia, the environmental acoustic conditions that have acted to maintain the system must be nearly universal. Clearly, extreme, traumatic sound levels do not meet this requirement. Alternatively, our review shows that low-level noise, both exogenous (wind and water) and endogenous (heart, myogenic, respiratory, gnathosonic, etc.), are universal. In the presence of this low-level, broadband noise, then, the ear is tasked with essential behaviors such as identifying predator and prey (e.g., sound localization), as well as communicating. It shares these tasks with the other sensory systems, and –not surprisingly- these other systems have evolved to possess similar mechanisms to address the problem of signal and noise: During waking hours, human beings are exposed to a dense and complex stream of stimuli, engaging all sensory domains. Because cognitive systems are limited in their ability to process this wealth of sensory data, mechanisms for selection of a subset of this information are needed, and these mechanisms are studied in the Psychology and Neuroscience laboratories using selective attention tasks. Across sensory modalities, one core theme in theories of selective attention has been that of amplifying certain features or aspects of the sensory signal that are relevant for behavior, while suppressing others. This emphasis on the signal-to-noise ratio in sensory signaling is evident in competition (Desimone, 1996) and normalization (Reynolds and Heeger, 2009) theories of visual selective attention, as well as in models of auditory cortical re-tuning towards behaviorally relevant auditory frequencies (Weinberger, 2004). Across, modalities, physical stimulus characteristics and experience-based, low-level amplification mechanisms in the brain provide signals to facilitate sensory cortical processing regions based on stimulus salience, in a bottom-up fashion. In addition, top-down projections into sensory systems serve to enhance relevant signals and/or suppress unwanted information, that is, the noise.

A number of reviews (cf., Robles and Delano, 2008; Robertson, 2009; Guinan, 2010) have detailed the numerous studies, both physiological and behavioral, showing the importance of MOC action in reducing the effects of noise, and increasing the signal-to-noise ratio for target signals (cf., Nieder and Nieder, 1970; Winslow and Sachs, 1987, 1988; Kawase and Liberman, 1993; May and McQuone, 1995; Andéol et al., 2011). How this is accomplished in the auditory system was explained by Liberman et al. (1996), when they demonstrated that the MOC was responsible for the rapid adaptation (RA) seen in OHCs. RA, with a time constant in the range of 50–100 ms, reduces OHC responses to sustained stimulation, and is relatively larger with binaural, compared with monaural sound owing to the inclusion of both crossed and uncrossed MOC subgroups in the response. Given that ambient background noises tend to be longer in duration, or effectively continuous, while biologically-important signals are brief or transient, the MOC suppresses responding to the noise alone and increases the signal-to-noise ratio for the target.

While MOC-mediated RA is a function of a low-level reflex loop, ending with crossed MOC fibers terminating on OHCs, important new findings suggest that corticofugal neurons, descending from the auditory cortex to the medial olivary complex, put the MOC system and, as a consequence, the OHCs, under cognitive control. A growing literature suggests that selective attention tasks produce alterations in peripheral auditory system function (Puel et al., 1988; Dai et al., 1991; Meric and Collet, 1992, 1994; Giard et al., 1994; Strickland and Viemeister, 1995; Maison et al., 2001; Delano et al., 2007; Srinivasan et al., 2012). As a whole, these studies show that representation in the auditory CNS of attended signals is relatively larger than ignored signals, owing to an apparent suppression of unattended sounds. Delano et al. (2007) showed conclusively that this effect resulted from MOC fibers terminating on OHCs. Delano et al. measured both the auditory nerve compound action potential (CAP) and cochlear microphonic (CM) response to auditory signals in chinchillas during visual and auditory tasks. They reported that CAPs recorded during visual discrimination tasks were smaller than, were suppressed compared with, CAPs recorded during auditory discrimination. Importantly, they also found that the CM response increased during the visual task, indicative of an MOC modulation of OHC membrane conductance resulting in a decrease in OHC gain (Mountain, 1980).

The function played by the so-called “uncrossed” MOC tract connecting the two ears in auditory signal processing is less well understood. When sound is played to the contralateral ear, these efferent neurons suppress responding in the opposite ear. Recent work by de Boer and Thornton (2007) has provided data suggesting that the uncrossed MOC is also under cognitive control. They showed that that shifts in the focus of attention may alter the ability of contralateral noise to suppress click-evoked otoacoustic emissions.

A study by our laboratory (Srinivasan et al., 2014) also supported that the uncrossed MOC has an important function in selective attention. We presented the same, brief tones simultaneously to both ears, with the overall duration of the tones varied in each ear. We compared the amplitude of DPOAEs recorded in one ear, as we instructed the subjects to count the shorter tones presented to the (1) ipsilateral or (2) contralateral ear, or (3) to ignore both ears and respond to subtle changes in a visual grating stimulus. DPOAE amplitude varied significantly with shifts in intermodal attention and, more importantly, when the focus of attention was shifted from one ear to the other. These data suggest that, like the corticofugal-crossed MOC tracts, the uncrossed MOC is under higher-level attentive control and can function to reduce the salience of ignored signals as a way of increasing the detectability of target signals.

## The role of MOC: increasing the signal-to-noise ratio

Out of necessity, most physiological studies, including those of the MOC, focus on how systems function within tightly controlled experimental conditions. As such, infrequent consideration is paid to the selective variables that may have been involved in the evolution of the system. From an evolutionary biology perspective, “function” is an imprecise term, and refers to the myriad roles that may be played by a system in a variety of contexts. In order to define the biological role played by a system, we must first identify whether or not the stimulus conditions necessary to evoke a specific function are routinely found in nature. Clearly, MOC protection from noise trauma fails in this regard, as the necessary extreme noise conditions simply do not exist. Alternatively, given the wealth of data, and recent reports of the benefits of MOC feedback in reducing the deleterious effects of aging on the cochlea, MOC effects may very well represent an important exaptation, or evolutionary spandrel (Gould, 1997).

**Figure 1:**
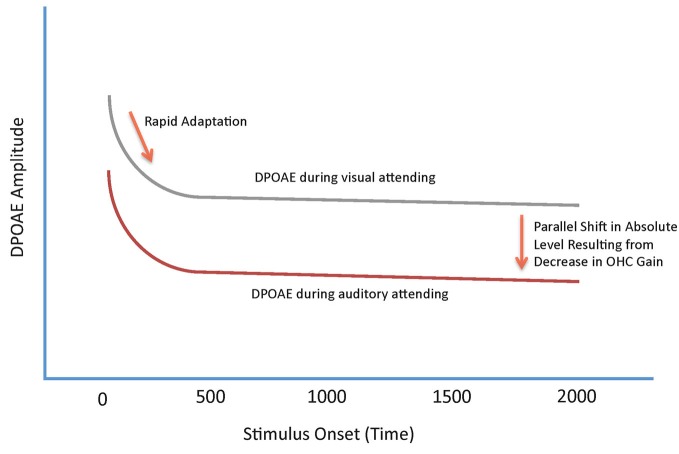
**Schematic summary of the effects of shifts in attention on DPOAE adaptation contours**. The two contours represent the amplitude of the DPOAE as a function of time from primary tone onset. The upper, gray contour, represents a DPOAE contour measured during a visual gradient discrimination task. The lower, red, contour represents a DPOAE measured during an auditory attending task. Both contours show a rapid decrease in overall level following stimulus onset produced by rapid adaptation (RA). DPOAEs measured during visual attending tasks are, on average, higher in overall level compared with DPOAEs measured during auditory attending tasks. The difference in DPOAE contours between the two functions is observed as a parallel shift in overall amplitude.

As described above, RA in OHCs is mediated by the MOC (Liberman et al., 1996). Thus, we considered DPOAE RA to relatively long-duration tones an ideal measure to characterize the role of the MOC in auditory selective attention in human listeners—the presence of RA in our data ensures that we are measuring MOC function (Bassim et al., 2003; Smith et al., 2012; Srinivasan et al., 2012, 2014). Initially, we hypothesized that changes in DPOAEs produced by shifts in the focus of attention would be observed as decreases in the slope of the RA function. Instead, what we have repeatedly observed across a number of different manipulations of attention, are* parallel* shifts in the absolute *level* of the DPOAE adaptation contour (Smith et al., 2012; Srinivasan et al., 2012, 2014). This effect is illustrated in schematic form in Figure 1.

That the MOC-controlled slope of the adaptation contour is unaffected by shifts in attention, and the entire function shifts only in absolute level, makes clear that two different, *concurrent* MOC processes are evident in the results; RA, which is entirely unaffected by attention, and a change in the gain of the cochlear amplifier to optimize the sensitivity of the cochlea, which is affected by the demands of the attention task. Both of the MOC phenomena observed in our data achieve the same result—increasing the signal to noise ratio for target signals via a reduction in responding to background noise; in the case of attention effects on cochlear gain, noise is defined as an unattended signal. By contrast, RA is a fundamental property of all sensory systems. It serves to decrease responding to sustained or repeated stimulation, preventing saturation and allowing for responses to new, transient stimuli. Because environmental noises, such as wind and water, are relatively long lasting, MOC RA suppresses the representation of these sounds at the level of the OHC, increasing the salience of new signals.

In studies of attention at the cortical or behavioral level, the effects of attending to an auditory signal are most typically observed as increases in the amplitude of the attended signal, compared with when that same signal is ignored (cf., Woldorff et al., 1987; Johnson and Zatorre, 2005; Kauramäki et al., 2007). Our DPOAE contours, on the other hand, are reduced in overall amplitude when listeners attend to the primaries (Figure 1; Smith et al., 2012; Srinivasan et al., 2012, 2014). We have explained this apparent paradox as being a consequence of the subjects’ attending to the primaries and, with the actual DPOAE being present in a different critical band, the DPOAE is suppressed by MOCs. Recent unpublished work in our laboratories measuring shifts in DP microstructure across different attended and unattended frequency bandwidths supports this notion (Srinivasan et al., unpublished). A similar effect has also been observed numerous times in psychophysical studies: For instance, in a simple threshold task where listeners are instructed to detect a specific frequency signal, thresholds for an unexpected tonal frequency are increased, and detection accuracy is decreased, with tonotopic distance from the expected frequency (cf., Dai et al., 1991; Strickland and Viemeister, 1995). Scharf et al. (1997) have demonstrated that this difference was reduced, or lost in patients with vestibular neuroectomy, where the MOC tracts to an ear are lesioned. This attentive process, like MOC neurons, is tuned and allows the listener to focus attention on one particular voice, or sound, at the exclusion of ignored sounds. This attentive tuning likely underlies the oft-mentioned “cocktail party” effect.

## Summary and conclusions

Our review argues that the biological role of the MOC system in hearing is to increase the signal-to-noise ratio for transient signals that are relevant for guiding behavior. This function results from two known processes that function concurrently, RA and selective attention, which both function to suppress environmental as well as task-irrelevant “noise”. This role is well supported by the extant physiological literature and describes functions of all MOC tracts into the ear, including their control by corticofugal neurons descending from auditory cortex. We argue that the environmental noise literature does not support that the MOC have evolved to protect the ear from acoustic trauma. We instead suggest that this noise-protective function might represent an evolutionary byproduct with beneficial consequences for the organism.

## Conflict of interest statement

The authors declare that the research was conducted in the absence of any commercial or financial relationships that could be construed as a potential conflict of interest.

## Footnotes

^1^Gould, with his collaborator R. C. Lewontin, applied the term “spandrel” to explain phenotypic traits that arise not because of their adaptive value, but as a consequence of other adaptations. In architecture, spandrels are any features, beit the triangular spaces over arched passageways in the Basilica di San Marco that inspired Gould and Lewontin, or the flat, rectangular spaces over a row of windows; the spandrels themselves were not the design feature, but are a consequence of, spaces “leftover” from design of the arches and, or windows. Regardless of how the spandrels have been adorned in the Basilica di San Marco, few would argue they themselves were designed specifically as artistic pieces. Gould and Lewontin likened architectural spandrels to evolutionary phenotypes that resulted, not because they have adaptive value, but as a consequence of the adaptive value of some related trait. Indeed, a spandrel might not confer adaptive value. Gould termed spandrels that conferred adaptive value “exaptations.” Here we argue that the protective effect of MOC action is an exaptation resulting from the suppressive nature of MOC function.

^2^jaxmice.jax.org
